# Lifestyle Changes and Industrialization in the Development of Allergic Diseases

**DOI:** 10.1007/s11882-024-01149-7

**Published:** 2024-06-17

**Authors:** Cevdet Ozdemir, Umut Can Kucuksezer, Ismail Ogulur, Yagiz Pat, Duygu Yazici, Sena Ardicli, Mubeccel Akdis, Kari Nadeau, Cezmi A. Akdis

**Affiliations:** 1 Institute of Child Health, Department of Pediatric Basic Sciences, Istanbul University, Istanbul, Türkiye; 2 Istanbul Faculty of Medicine, Department of Pediatrics, Division of Pediatric Allergy and Immunology, Istanbul University, Istanbul, Türkiye; 3 Department of Immunology, Aziz Sancar Institute of Experimental Medicine, Istanbul University, Istanbul, Türkiye; 4 Swiss Institute of Allergy and Asthma Research (SIAF), University of Zurich, Davos, Switzerland; 5 Department of Genetics, Faculty of Veterinary Medicine, Bursa Uludag University, Bursa, Türkiye; 6 Department of Environmental Studies, Harvard T.H. Chan School of Public Health, Cambridge, MA USA

**Keywords:** Allergy, Environment, Epithelial barriers, Microbiome, Toxicity

## Abstract

**Purpose of Review:**

Modernization and Westernization in industrialized and developing nations is associated with a substantial increase in chronic noncommunicable diseases. This transformation has far-reaching effects on lifestyles, impacting areas such as economics, politics, social life, and culture, all of which, in turn, have diverse influences on public health. Loss of contact with nature, alternations in the microbiota, processed food consumption, exposure to environmental pollutants including chemicals, increased stress and decreased physical activity jointly result in increases in the frequency of inflammatory disorders including allergies and many autoimmune and neuropsychiatric diseases. This review aims to investigate the relationship between Western lifestyle and inflammatory disorders.

**Recent Findings:**

Several hypotheses have been put forth trying to explain the observed increases in these diseases, such as ‘*Hygiene Hypothesis*’, ‘*Old Friends*’, and ‘*Biodiversity and Dysbiosi*s’. The recently introduced ‘*Epithelial Barrier Theory*’ incorporates these former hypotheses and suggests that toxic substances in cleaning agents, laundry and dishwasher detergents, shampoos, toothpastes, as well as microplastic, packaged food and air pollution damage the epithelium of our skin, lungs and gastrointestinal system. Epithelial barrier disruption leads to decreased biodiversity of the microbiome and the development of opportunistic pathogen colonization, which upon interaction with the immune system, initiates local and systemic inflammation.

**Summary:**

Gaining a deeper comprehension of the interplay between the environment, microbiome and the immune system provides the data to assist with legally regulating the usage of toxic substances, to enable nontoxic alternatives and to mitigate these environmental challenges essential for fostering a harmonious and healthy global environment.

## Introduction

**Fig. 1:**
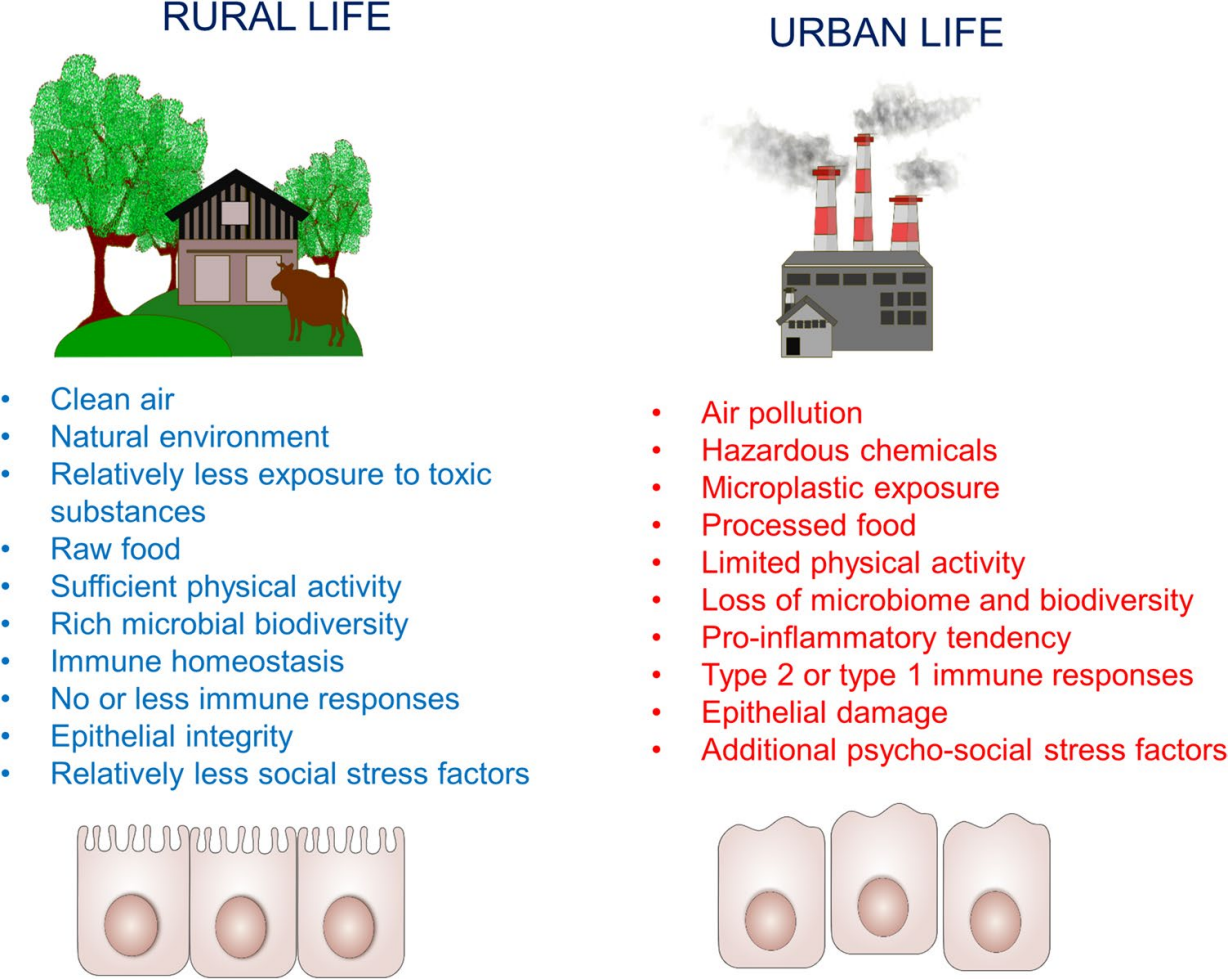
**Differences between rural and urban lives:** Westernization brings urbanization, which draw most individuals to live in cities instead or rural areas. These people consequently face air pollution, harmful chemicals, processed food and they have limited physical activity, all of which led to a loss in microbiome, predisposition to inflammatory conditions, and Th-2 biased immune responses. These mentioned factors collectively provide a tendency to epithelial barrier disruption, which underlies several inflammatory diseases including allergies, autoimmunity and neuropsychiatric conditions.

Allergic diseases have become increasingly prevalent and of great concern as they are reaching pandemic proportions [1–5]. Several hypotheses to explain these increases have been put forth [6, 7, 8•, 9••]. In addition to allergic disorders, certain inflammatory and chronic disorders have also increased in recent decades, mediated by epigenetics, environmental insults and the changing exposome [9••, 10]. Westernization and modernization during the last century has had many positive impacts on society. However, it has also brought with it loss of cultural habits-acculturation, isolation of individuals from society and nature, and adoption of a consumption-oriented fast and stressful life. Westernization has altered general practice and habits, sometimes in an unhealthy way [11]. Many Western diets contain calorically dense ultra-processed foods low in fiber and high in saturated fats, salt, and refined carbohydrates, and are associated with poor health outcomes including obesity, metabolic syndrome, and cardiovascular diseases [12]. In addition, Westernized diets increase risk for inflammatory bowel diseases, which is associated with gut microbial dysbiosis and a consequently a pro-inflammatory state [13]. As with Westernization, industrialization has also brought significant benefits to society. However, it also has adverse effects impacting global health. As examples, we are now commonly and frequently exposed to chemicals and pollutants such as food additives, detergents, exhaust fumes, and diesel particles, which effect the integrity of our epithelial barriers. Westernization is intricately linked with urbanization, potentially leading to reduced microbial biodiversity, (Fig. 1), [14]**.** Global warming has primarily been triggered by increased fossil fuel usage with industrialization, and this has led to increased frequency and severity of extreme weather events with adverse effects on planetary health. For example, due to co-action of Westernization and climate change conditions, pollination times are changed and prolonged, an increased variety of allergens are present for sustained periods, which collectively impact the course of allergic diseases [15–17].

**Fig. 2:**
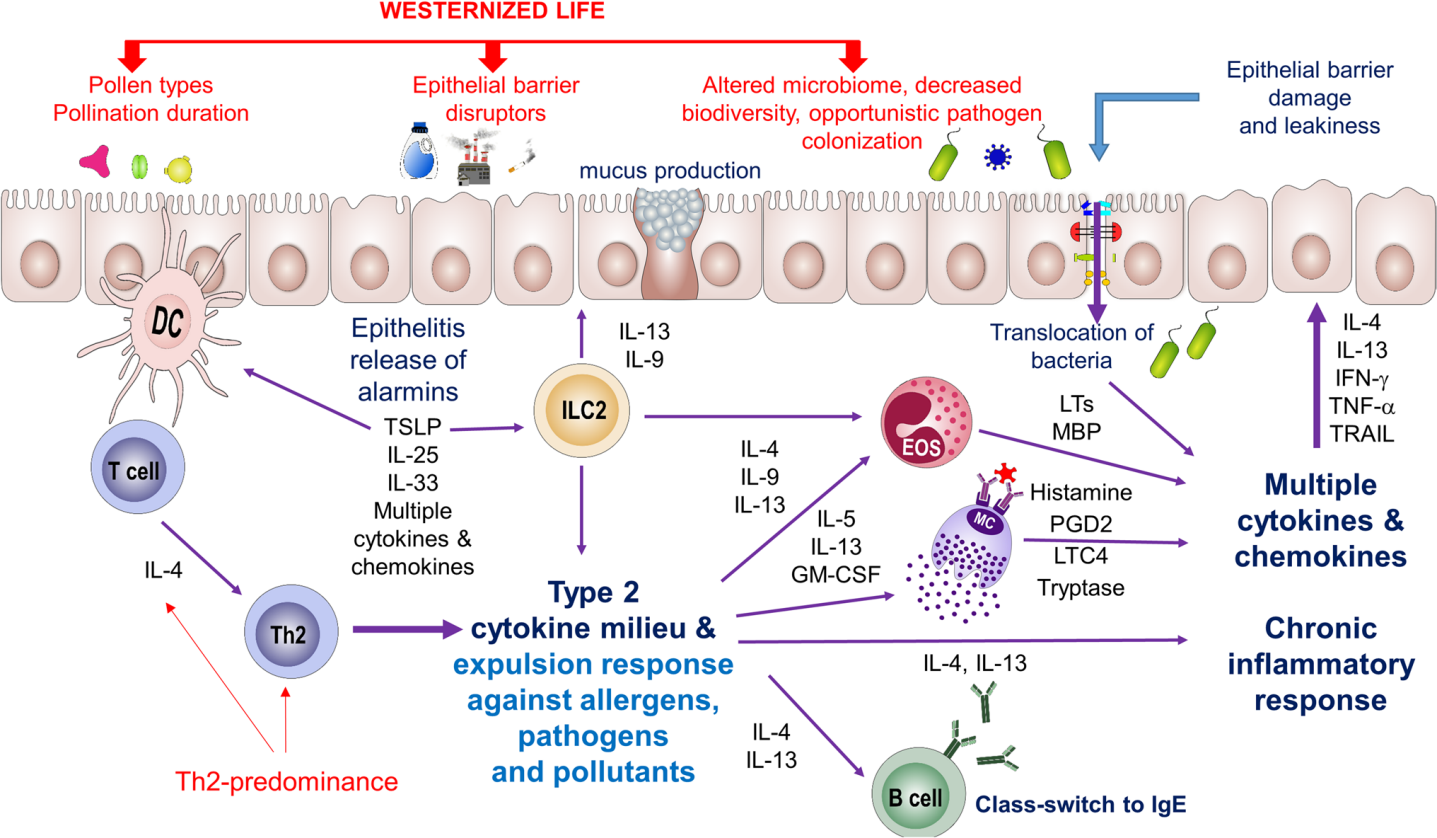
**Environmental contribution to allergic response:** The agents which are collectively termed as epithelial barrier disruptors harm epithelial barrier integrity, followed by initiation of epithelitis and release of alarmins such as IL-25, IL-33 and TSLP, all of which trigger immune responses. Alarmins secreted by epithelial cells activate dendritic cells (DCs) and type-2 innate lymphoid cells (ILC2) to induce Th2-type immunity. Disruption of epithelial barriers initiates microbial translocation, which contributes to a chronic expulsion response, triggered by rich milieu of Th2-type cytokines, supported by eosinophils and mast cells, by the production of mediators such as LTs, MBP and histamine, LTC4, PGD2 and tryptase. Commensal bacteria invade inside and beneath the epithelium and elicits cell migration and initiation of immune responses. When an allergen is captured by DCs , it is processed and presented to naïve CD4^+^ T lymphocytes. With the presence of IL-4 in the milieu, naïve CD4^+^ T cells are polarized to Th2-type T cells which can produce IL-4, IL-5, IL-9, IL-13, and GM-CSF, collectively termed as Th2-type cytokines. With the presence of Th-2 type cytokines, B cells class-switch to and produce allergen-specific IgE, which binds to specific IgE receptors present on mast cells, basophils and eosinophils; the effector cells of allergy. On the other hand, rich Th2-type cytokines activate and potentiate basophils and eosinophils. This phase is termed as sensitization, and if the same type of allergen is encountered for a second time, it directly binds to IgE antibodies present on effector cells of allergy and which immediately degranulate to release mediators such as histamine and leukotrienes. Activated immune cells including macrophages, DCs, mast cells, T and B cells, and ILCs migrate to the area and initiate a type-2 expulsion response with Th2 cells, IgE-producing B cells, ILC2, IL-4, IL-5 and IL-13 against opportunistic pathogens, commensals, allergens as well as pollutants. The opportunistic pathogens include *Staphylococcus aureus, Pneumococcus, Haemophilus* and *Moraxella*. IL-4, IL-13, IFN-γ, TNF-α, and TRAIL take part in the chronic expulsion response, leading to further epithelial damage. The inflammatory response together with translocated microbiome and microbial dysbiosis lead to defects in epithelium repair, and mis-closure of the barrier, which instigate a vicious cycle of leaky barriers and chronic inflammatory responses as well as microbial dysbiosis. Westernized life has important contribution in this process with introduction of epithelial barrier disruptors such as detergents, toxic chemicals, food additives, and pollution. Various pollens could be introduced to the environment due to careless plantation, while pollination times are altered, and periods are sustained due to global warming. An urban lifestyle leads to altered and limited microbiome, which could underlie increased Th2-type responses in industrialized countries.(DC: Dendritic cell, Eos: Eosinophil, GM-CSF: granulocyte colony-stimulating factor, IL: interleukin, ILC: Innate lymphoid cell, LTs: leukotrienes, M∅: Macrophage, MBP: Major basic protein, MC: Mast cell, PGD: prostaglandin, TRAIL: TNF-related apoptosis inducing ligand, TSLP: Thymic stromal lymphopoietin).

The ‘*Epithelial Barrier Theory*’ [9••] ultimately incorporates the former concepts; the “*Hygiene*”, “*Biodiversity*” and “*Old friends*” hypotheses in defining the impact of industrialization, urbanization and Westernized lifestyle on the epithelial barriers [6, 7, 8•, 9••]. Several disrupters of epithelial barrier integrity initiate a vicious cycle starting with epithelial cell death and damaged epithelial barrier. Consequent translocation of microbes, their toxins, toxic substances, as well as allergens to inter- and sub-epithelial areas trigger an inflammatory response in the epithelium named as “epithelitis”, with the release of alarmins, such as interleukin (IL)-33, thymic stromal lymphopoietin (TSLP) and IL-25 and many chemokines. This is followed by the migration of inflammatory cells in the area and chronic inflammation. This series of events lead to persistent leakiness of barriers and inflammation of the peri-epithelial areas, dysbiosis, opportunistic pathogen colonization and defective barrier healing, all of which could be an explanatory mechanism for allergic disorders and other many immune-related inflammatory diseases (Fig. 2), [9••,^10^,^18^].

A great deal of information is present thanks to the advances in the field; however, more intensive studies are required for better illumination of the link in between environmental changes, Westernization, and the pathogenesis. This review aims to present the most recent information, within the perspective of Western lifestyle and allergies.

## Allergic Diseases are on the Rise

Allergic diseases have become the trend topic of the last decades, on one side as the true prevalences of allergic diseases are markedly increased, on the other side, awareness of the general population and health professionals are also improved following the introduction of novel diagnostic methods [2, 4]. The epidemics of allergic disorders and this rise in prevalence of allergic disorders always tend to be explained and linked with the ‘Westernized lifestyles’, especially in industrialized and developing countries. Similarly, in addition to allergic diseases, autoimmune diseases and many other chronic inflammatory disorders are also on the rise, and their prevalences continues to increase in parallel to urbanization and industrialization [9••]. Supporting this concept, some evidence revealing a link between Westernization and an increase of allergic and other several inflammatory disorders has been accumulated during the last years [19, 20].

It is applicable that all allergic disorders show a kind of heterogeneity with variable outcomes, endotypes, phenotypes, theratypes and even regiotypes [21, 22]. Onset of these disorders, comorbidities, signs and symptoms, severity, treatment responses and diagnostic markers may vary. There is a dysregulated immune reactivity in which hypersensitivity and chronic inflammation prevail in the course of symptom-free times and exacerbations that may finally result with chronic changes as seen in remodeling and fibrosis. It is impossible to elucidate the cause of this increase only with the term ‘*atopy*’ and ‘*genetic background*’ especially in which twin studies could not supply adequate evidence [23]. In addition to genetic basis, there are numerous factors contributing to the pathogenesis of allergic disorders. Indoor and outdoor environmental conditions supplementary to exposure to infectious agents including bacteria, mycobacterium, viruses and parasites may exert either preventive or triggering roles. Furthermore, the mode of delivery, nutrition and composition of diet, previously used treatments such as antibiotics, vitamins, prebiotics, probiotics, and exposure to irritants, pollutants, and chemicals all defined as ‘exposome’ impact in this complicated pathogenesis [10].

## Westernized Lifestyle

Westernization is often regarded as an ongoing progression of globalization and modernization, which is taking place in several aspects, varying from economics, politics, social and cultural points of view. Although Westernization means taking positive characteristics of the developed world, sometimes, it also brings the discussion about loss or distortion of local habits, culture and individualization. ‘Westernized life’ sounds exciting, it is a lifestyle of isolation of individuals, and a fast, programmed, individualized and consumption-oriented life.

It is a major question of what Westernized life brings to us (Table 1). We started to live indoors, have been spending most of our times at homes and workplaces [24]. Exposure to chemical and biological agents including indoor allergens, pathogens and their related products additional to external physical causes, affects the risk of developing and course of allergic diseases. Indoor microbiome is continuously being ingested, inhaled and colonized the skin and mucous membranes [25]. There is an intimate relationship between these exposures, which can affect the epithelial barrier integrity and microbial biodiversity. Thus, a widespread understanding of the role of exposome is essential to plan a basis for designing and management of indoors [25].

**Table 1:** Alternations in lives following Westernization

**Topic**	**Before**	**After**	**Alterations**
Travel & transportation	Limited, infrequent	Frequent	Increased CO, CO_2_ emissions, air pollution
Traffic	Rare	Increased	Pollution, time-consumption, in-vehicle eating
Industry	Developing	Developed	Increased combustion, pollution, greenhouse effect
Housing conditions	Big surface area, high ceiling	Narrow places, lower ceilings	Increased allergen, mold, chemical exposure
Heating	Stove	Central and floor heating	Changes in indoor and outdoor air quality
Indoor temperature	Variable	Stable, overheated	Increased allergen, mold, chemical exposure
Illumination	Natural, daylight	Artificial	Changes in diurnal rhythm, hormonal imbalances
Family size	Large	Small	Decreased contact with elderly, less sibling numbers, less infections
Indoor pet ownership	Rare	Common, multiple	Exposure to pet antigens and allergens, infections of domestic animals, pet food allergens
Laundry	Washing by hand or washing machines	Automated washing machines, potent detergents, softeners	Frequent washing, exposure to detergent residues
Dishwashing	Washing by hands	Dishwashers, detergents and rinse aid	Frequent washing, exposure to detergent and rinse aid residues
House cleaning	Rinse cycle, infrequent	No-rinse cycle, frequent	Frequent cleaning, exposure to detergent residues
Diet	Traditional diet with limited consumption of processed and packaged food	Fast-food style, processed and packaged food	Less fiber, high-fat content and rich in refined carbohydrates, high salt intake
Drinking water	Tap water	Bottled water	Exposure to plastics, lack of essential minerals
Processed food	Unusual	Frequent	Exposure to emulsifiers, food additives, food dyes
Fruit and vegetable consumption	Seasonal, frequent	Non-seasonal, infrequent	Decreased exposure to novel food molecules including vitamins and anti-oxidants and microbiota found on plants
Coffee consumption	Infrequent	Excessive	Dehydration effect, exposure to sugar
Health facilities	Limited access	Easy access	Disease control, early diagnosis, overdiagnosis, overtreatment
Antibiotic usage	Infrequent	Frequent	Overtreatment, change of microbiota
Viral infections	Rare	Common	Increased treatment costs, lockdowns, loss of work and school days
Parasitic infections	Frequent	Rare	Improved clean water sources and sewage systems
Global warming and climate change	Unmarked	Highly marked	Extreme weather events, drought, floods, dust storms, wildfires
Clean water	Available	Limited access	Droughts, increased water-borne vectoral diseases,
Sea water	Clean	Polluted	Warming sea water, coral bleaching, sea level rise and loss of clean water, unhealthy sea species, extinction of species, microplastic pollution
Underground water	Clean, available	Polluted, limited	Contamination of chemicals, pesticides, loss of underground water sources, excessive usage for agricultural activities
Daily life	Active enrollment	Practical, sedentary	Insufficient physical activity, obesity, loss of social contact

In the Western lifestyle, housing conditions have changed especially in urban life as houses are now smaller in size, with low ceilings, open kitchens, stable temperature, more chlorine exposure, indoor and inner-city pollution, continuous cleaning need of the indoors, exposure to cooking and ovens, exposure to microplastic and nanoparticles, increased consumption of proceed/packaged food and so on. As the purchasing activities have markedly increased, especially after the COVID-19 pandemic through online-trading methods [26], there is a load of goods in our houses, which are serving as storage places for old and unused rotten and contaminated substances. With improved insulation methods as with plastic-welded glass systems, complete isolation is provided from the outside environment, and capturing humidity that may possibly increase indoor allergens especially mold contents [27, 28]. While previously, the houses were heated with appliances such as wood or coal stoves locally, today central heating systems are more common, enabling all areas of the house to be provided at the same temperature and sometimes overheated. Another variability seen is the usage of open kitchens as a trend of last years. Without sufficient ventilation, the steam and smoke emitted during the cooking activities affect the indoor air quality negatively with organic compounds and respirable food allergens [29]. Similarly, indoor air quality of working places is also important. In sick building syndrome due to exposure to volatile organic compounds, ozone, and lack of fresh air, employers develop symptoms such as headache, eye, nose throat irritation, fatigue, dizziness and nausea (Table 1), [30].

## Global Warming, Climate Change, Pollution and Health

Global warming is related to the alternations in the composition of the atmosphere because of human activities in combination with variations in the natural climate (Table 2). Westernization and industrialization have direct and indirect influences on the alternations that cause global warming and as a result climate change [31]. Following the industrial revolution, gas emissions from combustion of fossil fuels that can trap the heat of the sun and lead to an increase in global temperature, has increased, which could be termed as the greenhouse effect [31]. Global warming and climate change is a growing health concern, leading to extreme weather events resulting in increased risk of wildfires, hurricanes and thunderstorms, floods and desert dust storms. These natural disasters exert short- and long-term effects on planetary health, general health, as well as on many chronic diseases and health problems such as cardiac and respiratory-related morbidity and mortality [32]. The marked increase in global temperatures, resulting from the combustion of fossil fuels and the accumulation of greenhouse gases affect not only humans but also ecosystems worldwide [33]. Individuals living in populated areas like metropolitan cities could have aggravated health problems due to exposure to particulate matter (PM) as well as toxic gases [33]. Besides, heat stress could induce epigenetic alternations, which could collectively induce and up-regulate inflammation [34]. Due to global warming, critical changes may occur which may exert detrimental effects on health. For example, the ongoing changes in temperature extremes were shown to cause extended seasonal duration and increased pollen load for many pollen taxa in various places throughout the northern hemisphere [17]. For instance, common ragweed has been spreading in Europe because of warmer temperatures. Common ragweed grown under elevated CO_2_ levels in climate-controlled chambers produce pollen which elicit more robust allergic response both in vivo and in vitro models [16•]. This effect of ragweed pollen of increased allergenicity depends on the interplay of multiple metabolites, as metabolome analysis revealed differential expression of secondary plant metabolites in ragweed grown under elevated CO_2_ levels compared to controls [16•].

**Table 2:** Impact of weather changes and global warming on health and epithelial barriers

**Topic**	**Impact**	**References**
Greenhouse gas emissions	Increased atmospheric temperature Increased pollination	[16•, 31]
Extreme temperatures	Declined physical/mental health Heat stress Increased mortality/morbidity	[34]
Extreme weather conditions	Extinction of species Collapse of the ecosystem	[120]
Particulate matter and toxic gases	Declined health, epithelial barrier dysfunction	[33, 121]
Pollutants as ozone, particulate matter and nitrous oxides	Reduced epithelial functions Increased inflammatory conditions	[122, 123]
Altered epigenetic regulation	Chronic defects in epithelial barrier functions	[71]
Plant growth and pollination	Growth of species in irrelevant locations Altered sites and sustained duration of pollination	[16•, 17]
Melting glaciers, sea level rise Increased floods and droughts	Alterations in water sources Pollution of ground and surface water Increase in spread of infectious diseases	[124]
Wildfires	Toxic smoke, air pollution, increased drought, increased particulate matter Epithelial dysfunction	[35, 36]
Increased seasonal dust and sandstorms	Increased respiratory disorders Dose-dependent apoptosis/necrosis of airway cells	[40, 41]
Thunderstorms	Rupture of pollen grains Increased respiratory allergies	[38, 39]
Loss of biodiversity	Global warming modifies abundance of taxa, composition of communities, and ecosystem properties	[4, 8•]

The frequency of forest fires and wildfires has substantially increased in the last decade. In the summer season, forest fires often have natural causes, such as thunderstorm lightning. However, with more droughty and hot periods, the duration of the risk of forest fires is expected to increase. Population exposure and increased trend of outdoor activities in which insufficiently extinguished fireplaces or discarded matches were among the common causes. Additionally, socioeconomic factors such as the share of agriculture in the economy are other triggers of boosted forest fires. Early-life wildfire smoke exposure is related with immune dysregulation and lung function declines in adolescence [35]. There is epidemiologic and experimental evidence supporting that wildfire smoke exposure increases allergic predisposition and respiratory diseases, especially by exerting acute detrimental effects [36]. It has been suggested that epithelial integrity can be disrupted with downstream effects on T helper (Th)2 inflammatory or immune pathways due to wildfire smoke [37].

Extreme weather events impact allergic diseases. The 2016 epidemic of thunderstorm asthma in Melbourne was an extreme event with fatalities [38]. An intense number of bio-aerosol allergens ensues as the amount of inhalable allergens broken down by osmotic shock increases as thunderstorm accumulates pollens in lower layers of atmosphere [39]. Furthermore, dust storms arise from arid regions when robust winds carry huge amounts of loose sand and dirt into other geographical areas. Small sand particles can persist airborne for days and travel long distances [40]. Desert storms and meteorological variables such as ambient temperatures and PM pollution can impact pulmonary morbidity and mortality [41]. Emerging evidence implies that air pollution may contribute to the development of childhood asthma. In 18 European countries, more than sixty-thousand children were evaluated and one-third of asthma cases were linked with air pollution. After modeling, it was proposed that if the World Health Organization’s Air Quality Guidelines were followed, 11% of cases could have been prevented. [42]. It is evident that air pollution causes immune dysregulation by inducing oxidative stress, with epigenetic changes, altering mRNA expressions [43, 44].

## Westernization Influences Inflammatory Diseases by Alternation of Epithelial Barrier Functions

All abiotic or biotic environmental, ecological, or psychosocial factors affecting the living organisms constitute our exposome [45]. While general external exposome comprises climate, biodiversity, urban environment, and socioeconomic factors, specific external exposome includes allergens, microbiota, diet, tobacco, and pollutants. Besides, internal exposome contains individual metabolic factors, inflammation, and oxidative stress related to the host [10, 45–48]. There is a close interaction between all the organisms and the abiotic pools including the physical environment as ecosystems. Previous studies tried to illuminate the causes underlying the rapid increase in the prevalence of allergic diseases. The ‘*Hygiene*’ hypothesis claimed that alternations in the microbial environment could shape the development of the immune system, as recurrent microbial infections would trigger a Th-1 response instead of a Th2 response present in allergic individuals [6]. The ‘*Old friends*’ hypothesis reframed this idea and claimed the necessity of satisfactory exposure to microorganisms for a swift development of immunity [49]. Non-pathogenic commensal microorganisms that have existed throughout human presence were the source of immune regulatory signals that could avoid immune-mediated disorders [7]. The increase in allergies and inflammatory disorders following the Westernization was clarified by a requisition of fine-tuning of Th1 and Th2 responses through a microbial environment [50]. A complementary hypothesis to hygiene is the ‘*Biodiversity*’ hypothesis which proposes that a greater diversity of microbial species is a requisite for the delicate balance of the immune system. For the enrichment of microbiota, contact with the natural environment is compulsory [14]. Reduced biodiversity together with alternations in skin and gut microbiota composition could be linked with various inflammatory conditions encompassing allergic diseases [9••].

These three hypotheses concentrated on the role of microbiota in conditions comprising inappropriate immune responses and nominated that variations in the encountered microbial composition could be conclusive in disease development and pathology [9]. However, with today’s knowledge, these previous hypotheses are short of explaining the solid relationship between environmental changes, microbial triggers, alternations in the microbiome, and the increase in inflammatory disorders (Table 3). The allergy epidemic was noted in the 1960s, long after the introduction of water sanitation in western cities in the 1920s [2, 51]. Allergic asthma is still increasing in Asian and African cities with low hygiene [52]. When migrants from developing countries to prosperous places were investigated, a prompt increase in allergic and autoimmune diseases was noted [54–56]. The farming environment was long claimed to be protective for the development of allergic diseases and asthma. A previous study investigating farming Amish and Hutterite populations with very similar genetic backgrounds and lifestyles revealed that the Amish environment was protective against asthma due to traditional farming habits and thanks to a rich microbiome, compared with that of Hutterites. This was clearly reflected with the differences in prevalence of allergic diseases between the two communities. It is noteworthy that Hutterites farming methodology is highly industrialized [57]. In addition, cleaning and food habits of Amish still reflect the style of the seventeenth century with no usage of common detergents and packaged/processed food, whereas the cleaning and food habits of Hutterites are very similar to the next-door modern city. Accordingly, additional factors are required for a better explanation of the interrelation between diseases and the environment, such as urbanization, cleaning habits, packaged and processed food and diet alternations, use of antibiotics, caesarean birth, and air pollution collectively contribute to this increase, rather than public hygiene alone [58–60]

**Table 3:** The questions raised for previous hypotheses that are put forward in the epithelial barrier theory

**Status**	**Concerns of Epithelial Barrier Theory**
There is a sequential increase in prevalence of various allergic diseases such as seasonal allergic rhinitis, pediatric asthma, peanut allergy, Alpha-gal allergy [2]	Why do not these increases occur simultaneously? May correlate with the advent of usage of everyday substances
Water sanitation had started by 1910s, but allergy epidemics was marked by 1960s [51, 125]	Why a delay of 50 years? May correlate with the advent of the usage of everyday substances
Other chronic inflammatory diseases not related with Th2 immunity were also increased [9••, 13]	Disruption of epithelial barrier integrity may contribute to both Th1 and Th2 immune responses
Helminths, *Helicobacter pylori*, and hepatitis A virus are still around [126]	*Old friend* microorganisms are still with us
In the urban life a high number of microorganisms are ingested daily via respiration [127]	Urban life should bring protection, but not
Routine cleaning habits even involving use of antibacterial cleaners cannot exert sustained effect on levels of microbes in Westernized homes [128]	We cannot sterilize our homes as thought Disruption effect on the barriers of these cleaners may contribute the high increase of inflammatory disorders

Recently described metaexposome emphasizes the bidirectional effect of the environment on human subjects and their influence on all living systems and their genomes. Metaexposome deals with how influences changing an environment, especially through microbiota exposures, can affect health and disease over the lifetime [61]. The biotic and abiotic components are connected through nutrient cycles and energy sources. Living area, population density as well as Westernized urban life are the components of ecosystems. Among the Westernized lifestyle factors, physical activity, sleep behaviors, and diet in addition to habits such as smoking, alcohol, and drug use are the main contributors. Besides, psychological and mental stresses, inequality, cultural norms, networks, social capital, and home budget are the social dynamics affecting the exposome [10].

Intact skin and mucosal barriers are vital for the maintenance of tissue homeostasis as epithelial cells serve as the first-line defense of host tissues against external insults. For more than half a century, especially in Westernized life, humans have been exposed to many newly introduced noxious and toxic substances (Table 4). Loss of contact with biodiverse components of nature leads to loss of immune protective roles and has been proposed as a health risk, as humans are isolated from the soil through urbanization, through asphalt and concrete [14]. Functional disruption and formation of leaky epithelial barriers were claimed to be responsible for these inflammatory disorders [9]. The Epithelial Barrier Theory ultimately incorporates the former concepts; the “*Hygiene*”, “*Biodiversity*” and “*Old friends*” hypotheses in outlining the impact of industrialization, urbanization and Westernized lifestyle on the epithelial barriers (Table 3) [6–8, 9••].

**Table 4:** Environmental agents with capacity to disrupt epithelial barriers

**Agent**	**References**
**Pollution**	
Cigarette smoke	[129]
Diesel exhaust particles	[130, 131]
Ozone	[132]
Particulate matter	[133, 134]
Microplastics	[135]
Nano particles	[135, 136]
Volatile organic compounds	[29]
**Climate Change**	
Wildfires	[35–37]
Altered pollination	[16, 17]
Desert dust	[40, 41]
Deforestation	[137]
Greenhouse emissions	[137]
**Cleaning Agents**	
Detergents (Laundry)	[77]
Detergents (Dishwasher)	[78]
Household cleaners	[138]
Surfactants	[76]
Disinfectants	[139]
Shampoo, body cleaners	[76]
Household cleaners	[76]
Toothpaste	[139]
**Processed Food**	
Emulsifiers	[140]
Surfactants (polysorbates)	[81••]
**Allergens**	
Protease allergens (Mites, pollens, molds)	[141, 142]

## The Epithelial Barrier Theory

Nowadays, increased exposure to epithelial barrier-damaging agents is closely linked with the remarkable rise in many inflammatory disorders. The "*Epithelial Barrier Theory*" postulates that infectious agents, environmental toxins, pollutants and allergens all exert epithelial barrier-damaging effects by disrupting the epithelial barrier and inducing peri-epithelial inflammation, leading to the inflammation in epithelial barriers [9••,^15^,^62^–^67^] (Fig. 2). Following the loss of biodiversity, the dysbiotic microbiota can cross the damaged barrier and lead to the development of diseases including allergies, many autoimmune and other chronic conditions including Alzheimer’s disease, Parkinson’s disease, chronic depression, and autism spectrum disorders [9••].

Epithelial barrier disruption is linked with several factors. Genetic defects and mutations in barrier proteins such as filaggrin, loricrin, involucrin, and hornerin [68], and mutations in tight junction (TJ) proteins such as claudin and occludin all contribute to scarce integrity of epithelial barriers [69, 70]. Direct encounters with chemicals and pollutants in addition to other environmental factors could disrupt epithelial barriers [10]. The epithelial barrier as the first line of defense does not function properly after encountering epithelial barrier-disrupting agents, as demonstrated through biopsies of the affected tissues [69, 71–74]. More than 350′000 chemicals have been introduced to human lives without any major health concern [75]. Many of these substances ended up as pollutants that could damage body surfaces, skin, and mucosal epithelium, together with changes in the microbial biodiversity. Novel confounders have been demonstrated to damage epithelial barriers, such as chemicals, detergents, cleaners, shampoos, alcohol, smoking, food additives, food emulsifiers, microplastics, nanoparticles, ozone, diesel exhaust, PM, other pollutants and as well as the overall impact of the climate change that brings about increased exposure to toxic substances (Table 4). Several anionic surfactants and commercial detergents could decrease TJ barrier integrity in human keratinocytes [76]**.** Laundry detergents and detergent residues after rinsing could also disrupt TJ barrier integrity in human bronchial epithelial cells [77]. In addition, detergent residues from professional dishwashers have a remnant of a significant amount of cytotoxic and epithelial barrier-damaging rinse aid remaining on washed and ready-to-use dishware. The detergent toxicity was attributed to exposure in a dose-dependent manner up to 1:20,000 v/v dilution and alcohol ethoxylates present in the rinse aid were identified as the culprit components causing the epithelial inflammation and barrier damage, while the expression of genes involved in cell survival, epithelial barrier, cytokine signaling, and metabolism were all affected [76]. The laundry detergents and surfactants could induce eosinophilic airway inflammation in vivo through epithelial cell and innate lymphoid cell (ILC)-2 activation, which in turn induce IL-33 expression in airway epithelial cells through oxidative stress. Besides, detergent residues found in house dust are inhaled in daily life [79••]. Furthermore, epithelial barrier disruption is also related to inflammatory skin disorders. Laundry detergents and their main component, sodium dodecyl sulfate (SDS) impair the epidermal barrier of human skin both in vivo and ex vivo. Daily detergent and particularly sodium lauryl sulphate exposure may cause skin barrier disruption and may contribute to the development of atopic diseases [80]. Similarly, as recently demonstrated, food emulsifiers, especially polysorbate 20 and polysorbate 80 exert detrimental effects on intestinal epithelial integrity. Even at concentrations lower than 0.1%, polysorbates induce a proinflammatory response in organs-on-a-chip and induced pluripotent stem cell organoids [81••].

Following injury of epithelial cells by environmental disruptors, alarmins encompassing IL-25, IL-33, and TSLP elicit an inflammatory response in epithelial tissues, termed epithelitis. Epithelial cells and antigen-presenting dendritic cells cooperatively trim innate and adaptive arms of immunity. In contact with pollutants, allergens, and products of microbes, airway epithelial cells recognize antigens by pattern recognition receptors like Toll-like receptors and C-type lectin receptors. Through the NF-κB signaling pathway, airway epithelial cells produce cytokines such as IL-1β, IL-25, IL-33, granulocyte–macrophage colony-stimulating factor (GM-CSF), and TSLP, and also several chemokines (CCL2, CCL20), all of which collectively outline immune skewing and activation, and recruitment of other immune cells to the inflammation site [82, 83]. Inflammation in the affected epithelial barriers activates epithelial cells which in turn open their barriers and could lead to a leaky epithelial barrier [69, 72, 84] , (Fig. 2). A leaky barrier enables the microbiome's translocation from the periphery to inter-epithelial and even deeper sub-epithelial areas [18].

**Fig. 3:**
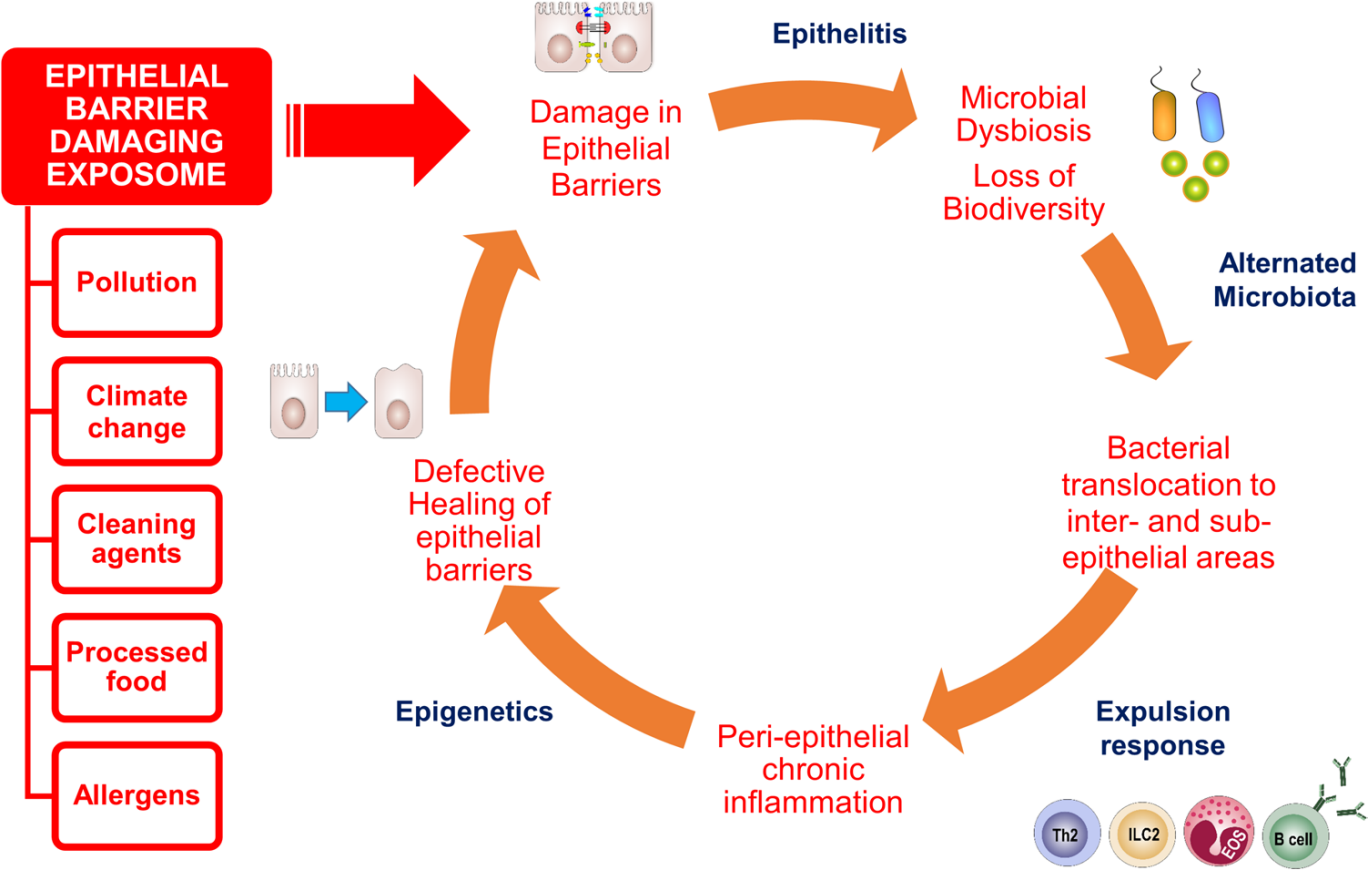
**The vicious circle of chronic epithelial barrier dysfunction:** Disruption of epithelial barriers are induced by exposome and damaging agents, which is facilitated by genetic defects in barrier-related molecules. Chronic inflammation in the peri-epithelial area leads to chronic, defective epithelial barrier healing and aggravates the damage. Epigenetics play role in defective barrier healing capacity which in turn leads to epithelial barrier damage and is termed as epithelitis. Then, loss of biodiversity and microbial dysbiosis end up with translocation of microbiota to inter- end sub-epithelial areas. An expulsion response is initiated, leading to chronic inflammation in peri-epithelial area. Defective epithelial barrier healing continues with epigenetic regulation

The succeeding colonization of opportunistic pathogens and poor biodiversity of commensal bacteria termed microbial dysbiosis generates an immune response against opportunistic pathogens like *Staphylococcus aureus* [18, 85, 86]. A Th-2 expulsion response comparable with the eosinophilic expulsion response developed against helminths is generated, in which the epithelial barrier opening is indispensable. This expulsion response incorporates Th2 cells, IL-13, eosinophils, and ILC2s, all collectively persuading the leakiness of epithelial barriers. Microbiota translocate to inter- and sub-epithelial zones, and the unremitting expulsion response creates a prolonged inflammation in the peri-epithelial area, and a vicious circle of leaky barriers, chronic inflammation, and microbial dysbiosis initiates chronic dysfunction of epithelial barriers [18], (Fig. 3). With the ongoing local inflammation, in addition to opportunistic bacterial colonization, microbiota dysbiosis, impaired tissue regeneration, and remodeling, it can be speculated that migration of the inflammation to distant organs from the inflammatory subepithelial areas may play a focus in the exacerbation of various chronic inflammatory diseases in distant sites such as allergies, multiple sclerosis, and diabetes mellitus [10, 85, 87–102]. In other words, defective epithelial barriers and linked distant inflammatory responses could underlie both allergic disorders and systemic autoimmune and metabolic conditions [18].

The metabolism of the host, immune homeostasis and integrity as well as functions of the epithelial barrier have robust links with microbiota [103]. Epithelial barrier damage and local inflammation are prevailed by microbial translocation with the presence of environmental agents to the sub-epithelial zones, and the initiation of a type-2 expulsion response [9, 10, 53, 104, 105]. A healthy microbiota could avoid the colonization of the pathogenic microbes in addition to the regulation, and improvement of epithelial barrier functions [106]. In tissues with a defective barrier, type 2 expulsion responses are initiated against commensal microorganisms together with pathogens [107]. *Staphylococcus aureus* is the most prevalent bacteria that colonizes the damaged epithelium of the skin and the respiratory system [108]. On the other hand, beneficial microbiota supports the healing and conservation of epithelial barriers [109]. Resident microbiota produces metabolites such as short-chain fatty acids, vitamins and tryptophan metabolites could contribute to the integrity of epithelial barriers [91, 110, 111].  In addition, fungal communities termed as mycobiota could have importance in the maintenance of the barrier health [112, 113]. The abundance or low expression of certain bacteria may be informative about disease development risk or protection. A relatively low abundance of *Bacteroidetes* was presented in children with food allergy [114]. *Acinetobacter* existence on the skin was accredited as “protective” against sensitization to allergens [115].

A crosstalk between epithelium, microbiome, and immune responses is responsible for both defense functions and the homeostasis of the epithelial barriers [116]. Both type-1 and type-2 immune responses beneath the epithelial barrier have the capacity to open the TJs [71, 72, 84]. IL-4 and IL-13 produced by CD4^+^ Th2 cells and ILC2 drive the opening of the TJ of epithelial barriers [71, 84]. IL-13 alone was revealed to have the capacity to open TJs [117]. On the other hand, Th1 responses and type-1 interferons could be triggered in keratinocytes via pattern recognition receptor activation and can disrupt the epithelial barrier integrity, as revealed in the skin [118]. Keratinocytes can neutralize pathogens directly and indirectly by activating other immune cells [119]. Following Major Histocompatibility Complex II upregulation on Langerhans cells and keratinocytes, leukocyte trafficking into the skin could lead to an immune cross-talk with T cells to develop sustained immune responses [115, 118].

## Conclusion

Westernization has two faces; one side eases lives with the advancement of technology, and a progressive health research provides better understanding of the pathogenesis of a great variety of diseases and supports advanced diagnosis and better care of patients by novel therapy options. On the other hand, environmental aspects such as pollution, global warming and associated alternations are influential in the development and exacerbation of many inflammatory disorders including allergic diseases caused by exposure to harmful chemicals, an increase in pollen burden, all of which collectively acts and disrupts the integrity of epithelial barriers. There is a need to continue research into the epithelial barriers to advance our understanding of the factors and molecular mechanisms associated with “leaky epithelial barriers”. Experimental models should be developed and validated to monitor the role of toxic substances on the development of leaky epithelial barriers. Possible strategies for the prevention, early intervention, and development of novel therapeutic approaches to reduce diseases associated with a disrupted epithelial barrier should be developed. Therefore, while obtaining advancements in life, sufficient attention to environmental aspects should be paid. We have only one planet to live on, it is our responsibility to take care of the environment, to limit some catastrophic disorders, which would be hard to cope with. 
